# Enhancing operational decision-making in hydrocarbon exploration drilling using machine learning for gas data interpretation

**DOI:** 10.1038/s41598-026-57038-8

**Published:** 2026-06-13

**Authors:** Gil Marcio Avelino Silva, Frederico Custodio Vieira dos Santos, Fernando Pellon de Miranda, Ygor Rocha, Igor Viegas Alves Fernandes de Souza, Janaina Andrade de Lima León, Bruna Souza da Silva, Rafael Nasser, Italo de Oliveira Matias, Sarah Barron Torres, Joelson Vialle Mathias da Silva, Moises Henrique Pereira, Francisco Fabio de Araujo Ponte

**Affiliations:** 1 Petróleo Brasileiro S.A, Rio de Janeiro, Brazil; 2 PUC-Rio, Rio de Janeiro, Brazil

**Keywords:** Mud-gas interpretation, Machine learning, Reservoir affinity, Drill bit metamorphism, Formation evaluation, PVT correlation, Energy science and technology, Engineering, Solid Earth sciences

## Abstract

Artificial intelligence is increasingly used to support decision-making during hydrocarbon exploration drilling, but mud-gas interpretation remains challenging because gas signatures are influenced by mud properties, drilling parameters, degassing efficiency, and Drill Bit Metamorphism (DBM). Here, we present a machine-learning-assisted workflow for assessing how well reservoir-fluid signals are represented in Advanced Gas (AG) measurements acquired while drilling. A multi-domain dataset from 104 Brazilian exploration wells was quality controlled, harmonized, and integrated with laboratory pressure-volume-temperature (PVT) fluid compositions and expert geological interpretation. Two predictive products were developed: Reservoir Affinity Curves, which estimate the similarity between AG signatures and reference PVT fluids using C2- and C2C-based targets, and a DBM Severity Curve, which quantifies drilling-induced thermal alteration using ethylene-related behavior and operational variables. Kernel Ridge Regression was selected as the primary deployment model because it produced stable, smooth, and interpretable depth-dependent predictions, whereas XGBoost and LightGBM achieved the highest numerical accuracy as benchmark models. The workflow distinguished intervals dominated by representative formation-fluid signatures from zones affected by DBM or other operational artifacts. This approach supports earlier fluid characterization, improves fluid-sampling decisions, and reduces interpretation uncertainty before laboratory results become available.

**Supplementary Information:**

The online version contains supplementary material available at https://doi.org/10.1038/s41598-026-57038-8.

## Introduction

The growing availability of geological and operational data, together with advances in computing power, has created new opportunities to apply machine learning (ML) in hydrocarbon exploration. Among these data sources, real-time mud-gas measurements remain underused despite their well-established potential to provide early indications of reservoir-fluid composition and hydrocarbon presence^1,2^.

Gas measurements acquired during drilling are highly sensitive to operational effects. Bit temperature, lithology, mud properties, and mechanical stress can distort gas signatures through Drill Bit Metamorphism (DBM), a thermomechanical process that alters hydrocarbon composition and can lead to misleading interpretations^1–3^. Robust validation and correction procedures are therefore needed to mitigate these distortions^4^.

Mud-gas data are often available before laboratory-based fluid characterization and can therefore support early reservoir evaluation. However, these measurements are often viewed as unreliable or only partially representative of formation fluids because of drilling-induced contamination, degassing inefficiencies, and operational variability^1,3^. These limitations highlight the need for structured methods that can extract meaningful information from real-time mud-gas data while systematically accounting for operational artifacts.

Despite advances in real-time gas analysis and ML applications, existing approaches rarely integrate gas interpretation with laboratory-based pressure-volume-temperature (PVT) correlation while explicitly quantifying DBM effects. To our knowledge, no previous study has combined similarity-based AG-PVT modeling with a dedicated DBM severity assessment using ML to distinguish native reservoir fluids from drilling-induced artifacts in real time.

This study presents and validates a machine-learning-assisted workflow that combines Advanced Gas measurements, drilling parameters, mud properties, and laboratory PVT compositions to improve the reliability and interpretability of real-time gas data during exploration drilling. The workflow produces two depth-dependent predictive outputs: the Reservoir Affinity Curve, which measures the similarity between chromatographic mud-gas signatures and laboratory PVT fluid compositions^5^, and the DBM Severity Curve, which assesses the extent to which thermal and mechanical effects influence AG measurements^1,2^. The process starts with data harmonization and operational validation in the WebMudlogging environment, followed by an exploratory data analysis step to examine relationships among gas signatures, drilling parameters, mud properties, and lithologic context. Machine-learning models are then used to generate predictive curves that support more robust interpretation and improved real-time decision-making while drilling.

By integrating these models into operational workflows, the proposed methodology improves early fluid characterization, supports safer and faster operational decisions, and provides a scalable and reproducible framework for machine-learning-assisted formation evaluation while drilling^4,6,7^. Overall, this work offers a scientifically grounded and operationally practical approach for transforming raw mud-gas data into actionable subsurface information.

### Background: gas chemistry and operational phenomena

Light hydrocarbons (C1-C5) are the most abundant gas components in drilling environments and typically remain in the gas phase under surface temperature and pressure conditions. Gas chromatography separates these components according to their boiling points, which generally correlate with molecular weight; lighter molecules elute before heavier ones^6^.

Unsaturated hydrocarbons, such as alkenes and alkynes, are generally absent from native reservoir fluids because thermodynamic stability over geologic time favors their conversion to saturated hydrocarbons, primarily alkanes. However, alkenes may appear as contaminants introduced during drilling operations or as byproducts of thermal degradation of drilling mud^2,3^.

DBM refers to the thermomechanical alteration of drilling mud at the bit-rock interface due to localized overheating. Temperature increases controlled by lithologic hardness, bit design, weight on bit (WOB), and rotations per minute (RPM) can induce thermal cracking of oil-based mud and generate artificial gases enriched in unsaturated hydrocarbons, such as ethylene (ETHE) and propylene^1,2^. These DBM-related compounds, together with other non-hydrocarbon species, can substantially alter downhole gas composition. Identifying DBM signatures is therefore essential for distinguishing native formation hydrocarbons from operational artifacts and preserving the integrity of subsurface fluid interpretation^1–3^.

Operational parameters such as increased WOB and RPM are strongly correlated with elevated concentrations of DBM-related gases, reflecting mechanical stress and thermal loading at the bit-rock interface^1,3,5^. Ethylene is the most consistent chemical indicator of DBM and is frequently observed under conditions of excessive drilling-induced thermal alteration.

Advanced Gas (AG) refers to compositional gas measurements acquired during drilling that capture gases released from formation fluids into the drilling mud and transported to the surface with the cuttings. Light hydrocarbons (C1-C5) are generally quantitative and, when recorded under adequate quality conditions, can be correlated with laboratory gas-chromatography data from PVT samples^4–6^. In contrast, heavier components such as C6-C8 and aromatic hydrocarbons are typically qualitative and require careful interpretation because of degassing and adsorption effects^4^.

**Fig. 1:**
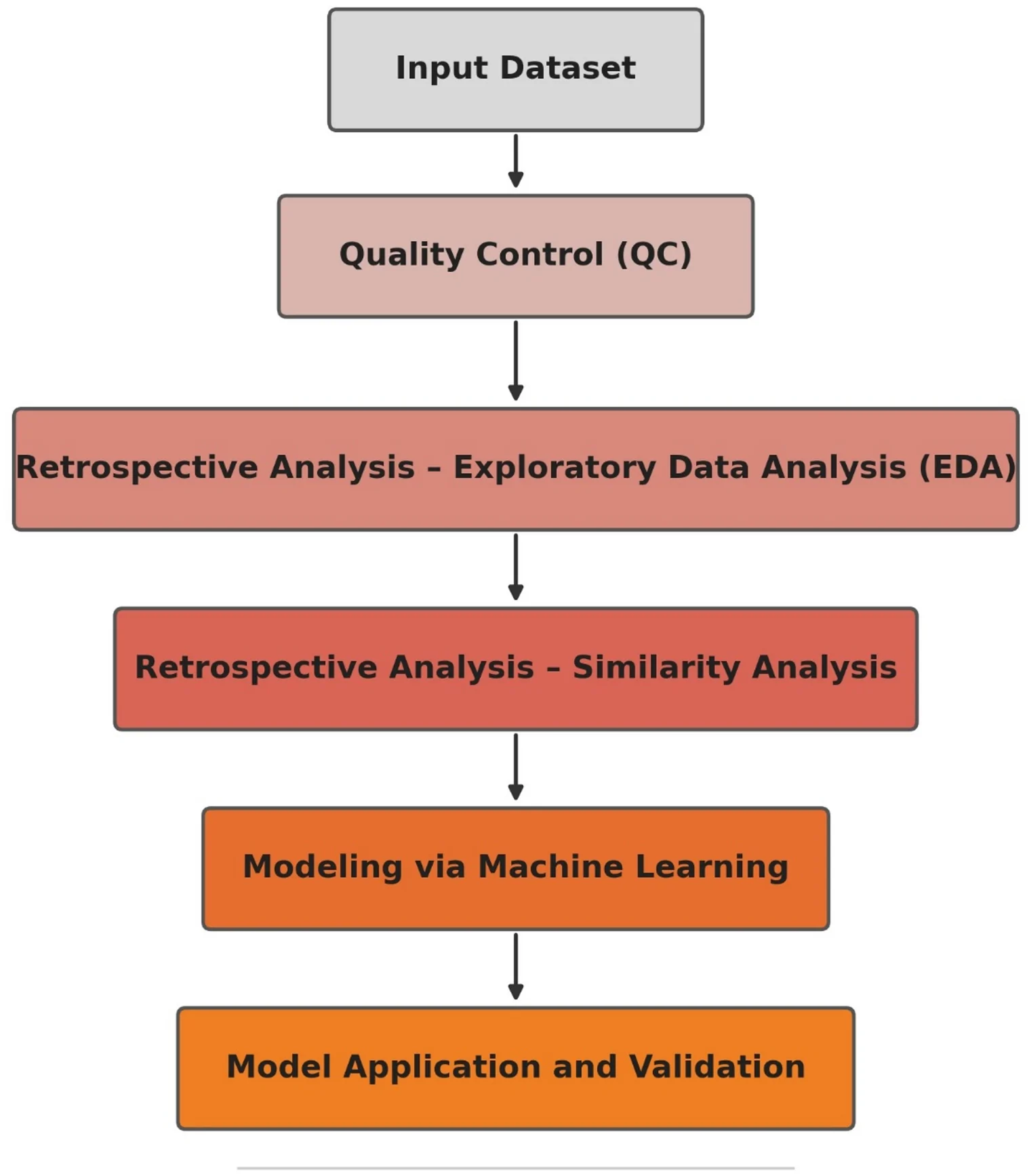
Workflow for data preparation, exploratory and similarity analysis, machine-learning modeling, and predictive application. The workflow begins with input datasets and quality control, followed by retrospective exploratory analysis, similarity analysis, model development, and model application and validation.

**Fig. 2:**
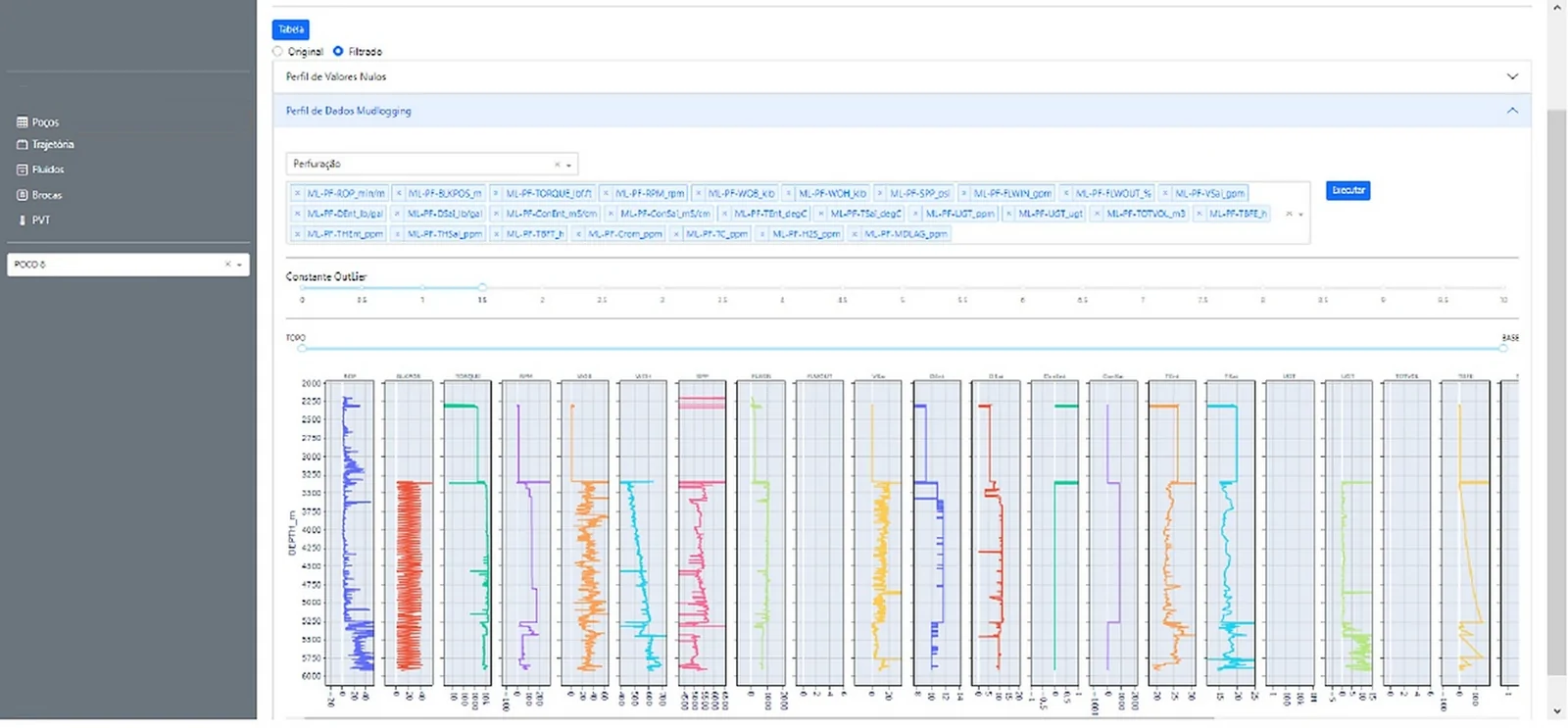
WebMudlogging interface used for inspection and validation of drilling parameters, gas logs, and PVT data. The platform supported traceable data review before machine-learning model development.

**Fig. 3:**
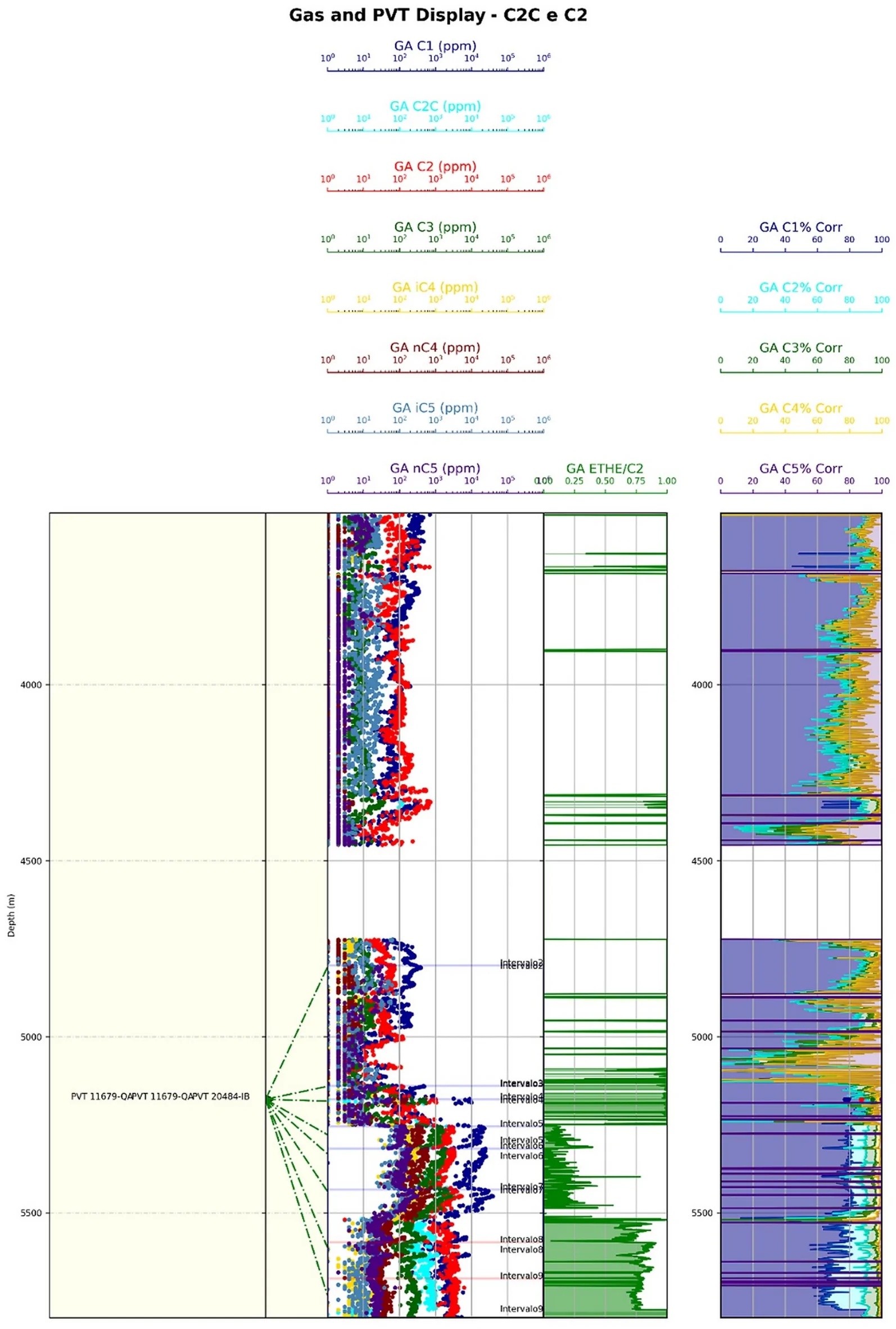
Example of AG, PVT, ethylene ratio, and similarity curves aligned by depth. The depth profile illustrates reservoir intervals and DBM-related artifacts used to support similarity analysis and supervised target construction.

PVT data are generated under laboratory conditions from downhole reservoir-fluid samples collected during open-hole logging and include heavier hydrocarbons (C7+). These analyses provide detailed fluid characterization across pressure, temperature, and volume conditions, enabling estimates of density, viscosity, saturation pressure, gas-to-oil ratio, compressibility, and shrinkage^4,7^. In this study, emphasis was placed on three AG-derived variables: C2 (ethane plus ethylene), C2C (ethane), and ETHE (ethylene). These variables are central to the similarity models and to the regression models used to generate the Reservoir Affinity and DBM Severity Curves (Figs. 1, 2 and 3).

## Results

### Model performance

**Fig. 4:**
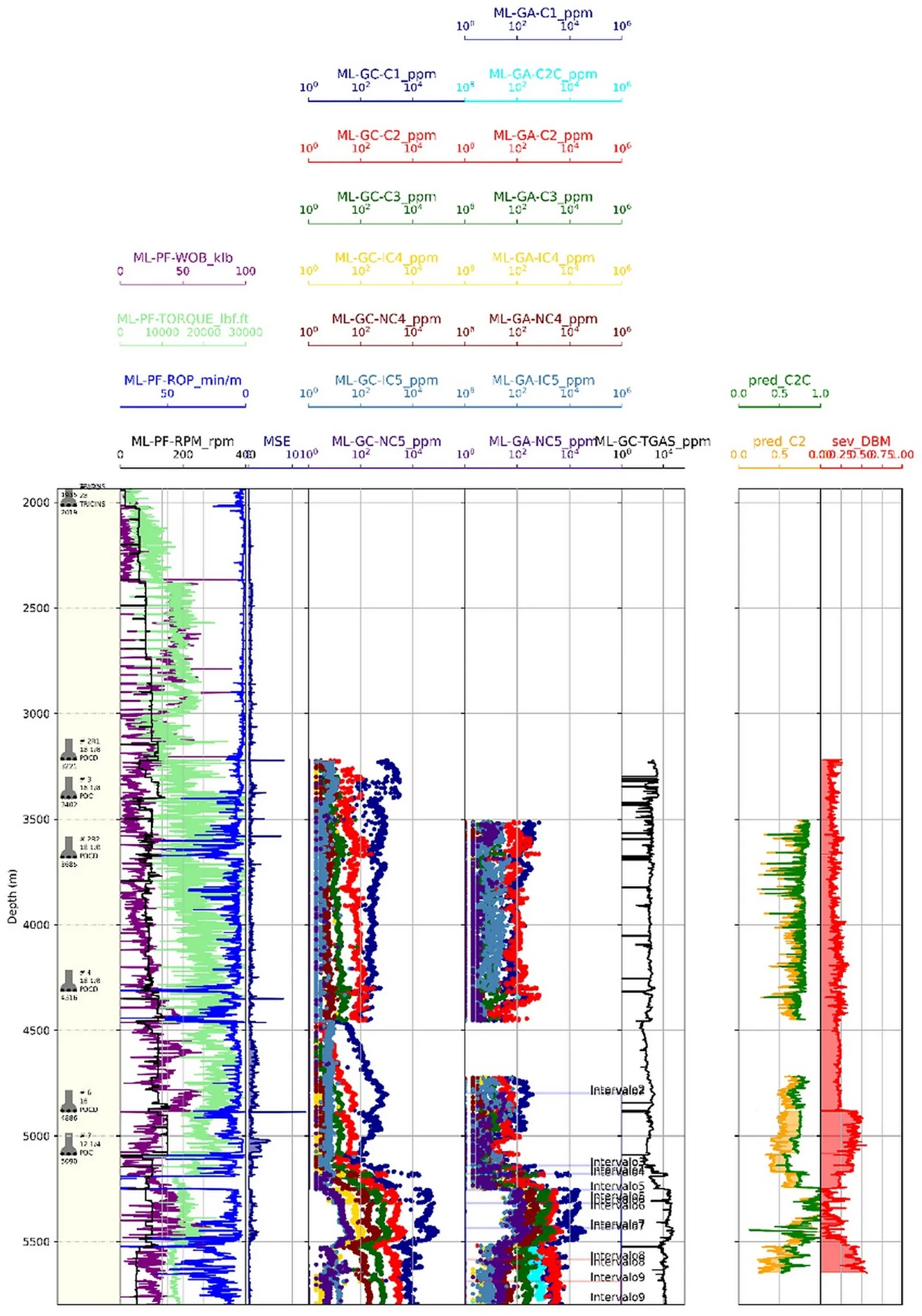
Model application in a representative well. Predicted C2 and C2C Reservoir Affinity Curves and the ETHE-based DBM Severity Curve were generated using Kernel Ridge Regression and plotted against depth with AG readings and PVT reference information.

The machine-learning models showed strong predictive performance for both Reservoir Affinity Curves (C2 and C2C) and the DBM Severity Curve (ETHE) (Fig. 4). Comparative metrics for Kernel Ridge Regression (KRR), XGBoost, LightGBM, and a feedforward neural network are summarized in Table 1.

**Table 1:** Performance comparison of Kernel Ridge Regression (KRR), XGBoost, LightGBM, and a feedforward neural network for predicting affinity curves (C2 and C2C) and DBM severity (ETHE).

Model	C2 *R*²	C2 RMSE	C2C *R*²	C2C RMSE	ETHE *R*²	ETHE RMSE
KRR	0.84	0.111	0.83	0.084	0.84	7.70
XGBoost	0.97	0.045	0.98	0.024	0.90	6.21
LightGBM	0.98	0.036	0.98	0.023	0.91	5.94
Neural network	0.84	0.112	0.81	0.090	0.91	6.13

Benchmark experiments using XGBoost, LightGBM, and a feedforward neural network were performed under the same well-level partitioning strategy and evaluation metrics (R² and RMSE). XGBoost and LightGBM achieved the highest numerical accuracy across all targets, with R² values reaching 0.97–0.98 and RMSE values as low as 0.023 for the C2C model. The neural network showed intermediate performance, whereas KRR produced moderately lower R² values (0.83–0.84) but remained consistent across all targets.

Although KRR did not produce the lowest RMSE, it was selected as the primary operational model because it provided greater stability, lower apparent variance across wells, and more interpretable depth-dependent predictions. These attributes are important for real-time deployment and expert-assisted decision-making in drilling environments. KRR also produced smooth learning curves with minimal evidence of overfitting, supported by closely aligned training and validation profiles.

The C2- and C2C-based affinity models achieved strong predictive performance (R² = 0.83–0.98 and RMSE < 0.10), supporting their use for estimating compositional similarity between AG signatures and laboratory PVT fluid compositions. The ETHE-based DBM Severity Model also showed strong predictive capability, especially for gradient-boosting models such as XGBoost (R² = 0.90), highlighting its sensitivity to drilling-induced thermal alteration.

In well-characterized intervals, the affinity and severity curves closely matched expert interpretation. Minor deviations were observed in deeper sections, mainly in the C2-based affinity curve, probably because of thermal alteration and mud-gas mixing. These deviations did not materially affect operational interpretation in the representative cases evaluated. Although boosting-based models offer higher numerical accuracy and should be further evaluated, KRR remains the most balanced model for consistency, explainability, and deployment readiness.

Figure 4 shows a representative model application, including the predicted C2 and C2C Reservoir Affinity Curves and the ETHE-based DBM Severity Curve generated with KRR. High-affinity intervals (similarity > 0.7) indicate that gas composition is strongly representative of formation fluids, whereas DBM-affected zones are characterized by elevated ETHE concentrations and reduced affinity. These curves support operational interpretation by distinguishing intervals dominated by native reservoir signatures from intervals influenced by mud-gas mixing, thermal degradation, or mechanical drilling artifacts.

### Feature importance

**Fig. 5:**
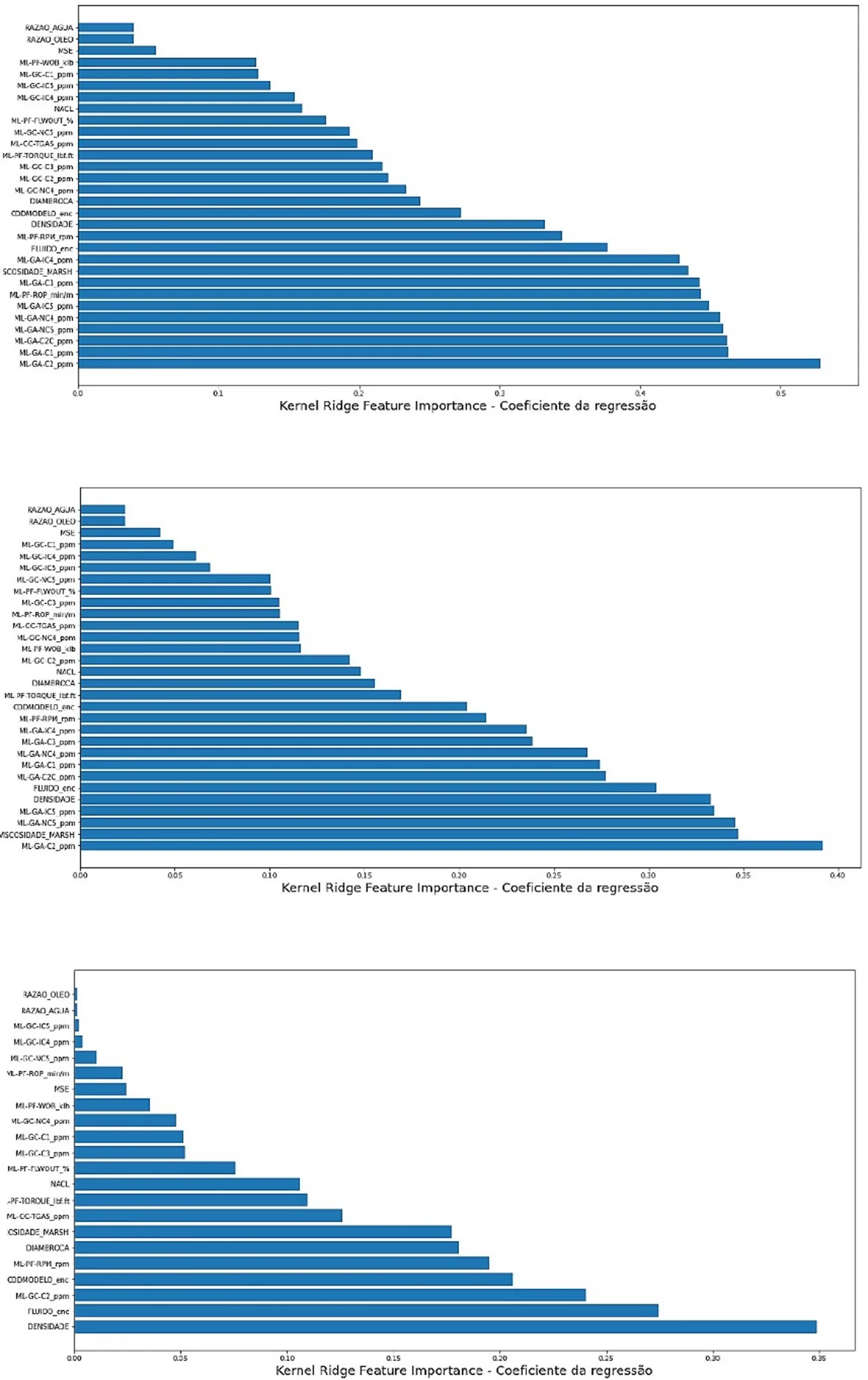
Permutation-based feature importance for the C2C affinity, C2 affinity, and DBM severity models. Rankings indicate the relative contribution of gas, mud, and operational variables to each predictive target.

Feature importance was evaluated using a model-agnostic permutation approach. For each feature, values were randomly permuted along depth while all other inputs were kept unchanged, and the resulting increase in prediction error (RMSE) was measured. Final rankings for the C2C affinity, C2 affinity, and DBM severity models are shown in Fig. 5.

Gas-related variables, especially AG channels, were the most influential predictors of fluid compositional affinity. In contrast, mud-related and operational variables had greater influence in the DBM Severity Model. Although Mechanical Specific Energy is operationally recognized as an indicator of drilling efficiency, it showed low permutation importance for the C2, C2C, and ETHE targets. This suggests that, in this dataset, affinity and severity responses were more strongly governed by gas signatures, mud properties, and selected drilling parameters than by energy-based indicators alone.

For both affinity models, GA-C2 consistently emerged as the dominant predictor. The DBM model, by contrast, highlighted the stronger role of mud properties and mechanical interaction variables, including mud density, mud type, bit diameter, and selected conventional gas variables.

### Interval classification during drilling

In this study, the term reservoir quality does not refer to petrophysical attributes such as porosity, permeability, or fluid saturation. Instead, it refers to the degree of compositional similarity between real-time AG readings and laboratory PVT fluid signatures. For operational interpretation, each depth interval was classified into one of three categories based on the affinity curves and the ETHE-derived DBM severity indicator.

High-affinity intervals are zones in which AG composition closely matches the laboratory PVT fluid profile (similarity score > 0.7), suggesting potential fluid representativeness and limited operational interference. Transitional intervals show fluctuating similarity values due to lithologic transitions, mud-circulation effects, or moderate operational artifacts. DBM-affected intervals are characterized by elevated ETHE concentrations and reduced affinity, indicating thermally induced compositional distortion associated with DBM.

This classification is intended as an operational decision-support tool during drilling. It is not a petrophysical assessment of reservoir quality, but rather an evaluation of how well the gas data chemically represent native formation fluids.

### Learning-curve behavior and overfitting assessment

**Fig. 6:**
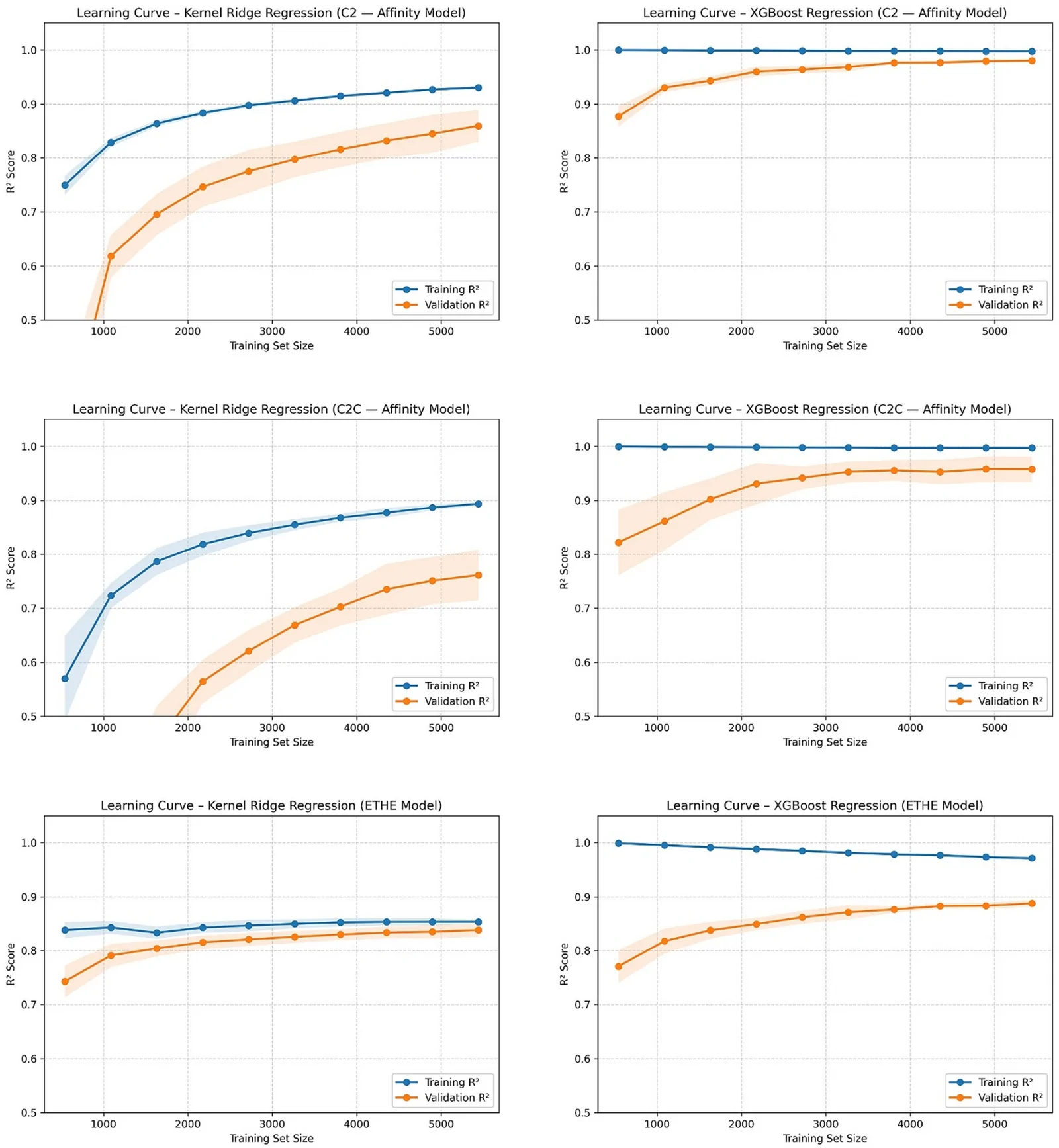
Learning curves for the three predictive targets (C2, C2C, and ETHE) using Kernel Ridge Regression and XGBoost Regression. Each panel shows training and cross-validation scores as a function of training-set size, with shaded areas representing one standard deviation across well-based cross-validation folds.

Learning-curve analysis showed that both KRR and XGBoost had strong generalization capacity under the well-level cross-validation framework (Fig. 6). For all targets, the training and validation curves remained closely aligned and the variance bands were narrow, indicating limited overfitting. XGBoost showed the lowest validation errors and the smallest uncertainty envelopes, whereas KRR provided smoother convergence and stable behavior across sample sizes. These patterns support the robustness of the methodology and its suitability for real-time operational use.

## Discussion

The results demonstrate that AG data acquired while drilling can be transformed from a largely qualitative monitoring signal into a quantitative decision-support product when integrated with laboratory PVT reference information, drilling parameters, mud properties, and machine-learning models. This integration is important because mud-gas composition is affected not only by reservoir-fluid composition but also by gas release, surface extraction, drilling-fluid interaction, and mechanical conditions at the bit. The proposed workflow therefore addresses a central limitation of conventional mud-gas interpretation: separating native formation-fluid signals from operational artifacts during real-time drilling.

The Reservoir Affinity Curve is the study’s main scientific contribution. Conventional gas interpretation often relies on individual ratios or visual comparison of gas profiles. These approaches are useful for screening but may not capture the multivariate structure of light-hydrocarbon distributions. By defining a depth-dependent similarity target between AG and PVT compositions, the proposed method turns the comparison between surface gas and reference reservoir fluids into a reproducible numerical framework. This makes it possible to evaluate similarity continuously along the well path rather than only at discrete sampling points. The approach is consistent with previous work showing that mud-gas data can support early fluid characterization when acquisition quality and operational context are properly controlled^6,7^.

The C2- and C2C-based affinity models provided complementary information. C2 represents the combined ethane-plus-ethylene signal and is therefore sensitive to both reservoir-fluid composition and thermal alteration. C2C isolates the ethane contribution and may provide a more conservative indicator of native hydrocarbon affinity where ethylene enrichment is present. The simultaneous interpretation of both curves is therefore preferable to relying on a single gas component. Intervals where C2 and C2C affinity are both high are more likely to represent formation-fluid signatures, whereas divergence between them may indicate DBM influence, mud interaction, or other operational effects.

The DBM Severity Curve adds a second interpretive layer by explicitly quantifying the likelihood that gas composition has been altered by drilling-induced thermal effects. Previous studies have shown that DBM can generate artificial gas signals and that unsaturated hydrocarbons, especially ethylene, are useful markers of this process^2–5^. The present results are consistent with that interpretation: ETHE-related behavior, together with mud and mechanical variables, provided a practical basis for identifying intervals where gas composition should be interpreted with caution. This is operationally important because DBM-affected intervals can mimic changes in reservoir-fluid character and may lead to inappropriate sampling or testing decisions if they are not recognized.

The feature-importance results provide additional geological and operational insight. AG variables dominated the affinity models, confirming that compositional information is the primary driver of AG-PVT similarity. In contrast, the DBM model assigned greater relevance to mud properties, bit diameter, mud type, and selected conventional gas variables, suggesting that drilling-fluid behavior and bit-rock interaction exert a stronger influence on thermal-artifact generation. The low permutation importance of Mechanical Specific Energy does not necessarily mean that mechanical energy is irrelevant to DBM; in this dataset, its contribution may be partly represented by correlated variables such as WOB, RPM, torque, bit diameter, mud type, and density. This interpretation is consistent with the multivariate nature of drilling-induced thermal effects.

The comparison among KRR, XGBoost, LightGBM, and a feedforward neural network highlights an important tradeoff between predictive accuracy and deployment suitability. Gradient-boosting models achieved the lowest RMSE and the highest R² values, showing a strong ability to capture nonlinear patterns in the dataset. However, KRR produced smoother predictions, showed less variation across wells, and yielded more stable learning curves. These qualities are valuable in real-time operational settings, where abrupt model fluctuations can reduce user confidence and make expert interpretation more difficult. For this reason, KRR was selected as the primary deployment model, while XGBoost and LightGBM remain strong candidates for future use, particularly if combined with uncertainty quantification and rigorous external validation.

The well-level splitting strategy was essential for obtaining a realistic assessment of model generalization. Depth samples from the same well are strongly correlated because they share geology, drilling practices, mud system, sensor configuration, and operational history. Random sample-level partitioning would therefore overestimate predictive performance by allowing the model to learn well-specific patterns that also appear in the test set. By separating entire wells into training and testing subsets, the evaluation more closely reflects the intended operational use case: applying the trained workflow to new wells with related but not identical geological and operational conditions.

From an operational standpoint, the combined interpretation of affinity and DBM severity can support several drilling decisions. High-affinity intervals with low DBM severity may be prioritized for fluid sampling, pressure testing, or closer geological evaluation. Intervals with reduced affinity but elevated DBM severity can be flagged as potentially contaminated by drilling-induced gas generation, reducing the risk of misclassifying formation fluids. Transitional intervals can prompt additional quality control, integration with lithologic observations, or comparison with petrophysical logs. In this sense, the workflow does not replace expert judgment but adds a quantitative layer that supports faster and more consistent multidisciplinary interpretation.

The integration with WebMudlogging is also significant. Real-time interpretation requires more than an accurate model; it requires traceable data, quality-control procedures, visualization tools, and expert interaction. The dashboard environment allowed gas curves, operational parameters, mud properties, lithologic indicators, PVT references, and model outputs to be interpreted together. This integration helps avoid isolated model use and supports a workflow in which geochemists, drilling engineers, and operations teams can jointly evaluate whether observed gas changes are geologically meaningful or operationally induced.

Several limitations should be noted. First, the dataset is proprietary and focused on Brazilian sedimentary basins, so the models should not be assumed to generalize to all basin types, mud systems, drilling technologies, or reservoir-fluid families without recalibration. Second, the PVT data used for calibration are discrete laboratory references, whereas AG measurements are continuous surface observations collected along depth and influenced by lag correction, gas-extraction efficiency, and mud circulation. Third, although ETHE is a strong indicator of DBM, it is not unique; contamination, mud additives, thermal degradation away from the bit-rock interface, and analytical effects may also influence unsaturated-gas signatures. Fourth, model performance depends on the quality and completeness of operational variables, and missing or inconsistent sensor data may reduce reliability. Finally, this analysis is retrospective, and prospective validation during active drilling campaigns is still needed before full automated deployment.

The thresholds used for interval classification should therefore be interpreted as decision-support criteria rather than universal cutoffs. For example, the high-affinity threshold of 0.7 provides a practical screening value in the present dataset, but optimal thresholds may vary with basin, fluid type, mud system, and operational objective. Future work should incorporate probabilistic calibration, confidence intervals, and uncertainty visualization so that users can distinguish robust predictions from intervals requiring expert review. This is particularly important for high-impact decisions such as downhole fluid sampling or changes in drilling parameters.

The broader implication of this study is that mud-gas geochemistry can be linked more directly to reservoir evaluation when combined with validated operational data and machine-learning models. The methodology provides a bridge between real-time drilling data and post-well laboratory characterization, enabling earlier insight into fluid representativeness and potential drilling artifacts. Future development should focus on prospective validation, integration with petrophysical logs and MDT/wireline sampling results, testing in high-pressure/high-temperature and deepwater settings, and deployment of model-monitoring routines to detect model drift as new wells and operational conditions are added to the database.

## Methods

### Dataset and materials

The dataset included 104 exploration wells distributed across Brazil’s major sedimentary basins and covered a broad range of geological settings and operational conditions. It integrated drilling parameters, bit specifications, mud properties, AG compositions, laboratory PVT fluid data, and expert geological interpretation. The main data categories included drilling data (depth, rate of penetration, torque, RPM, WOB, flow out, and conventional C1-C5 gas readings), bit specifications, mud properties, AG compositions, PVT fluid compositions, and expert interval classifications. The full list of input features is provided in Supplementary Table S1.

The workflow was implemented in Python using established scientific libraries. Scikit-learn was used for machine-learning algorithms and performance metrics, including KRR^8^. Pandas and NumPy were used for structured data manipulation and numerical computation, and Matplotlib was used for exploratory and diagnostic visualization. XGBoost and LightGBM were included as benchmark algorithms. The overall workflow included data preprocessing, exploratory analysis, similarity assessment, machine-learning modeling, and predictive-curve generation (Fig. 1).

### Data quality control and preprocessing

All drilling and gas readings came from Petrobras’s official mudlogging database and were reviewed, corrected, and validated at the well site by operational specialists using the WebMudlogging platform (Fig. 2). Statistical normalization, categorical encoding, and other automated transformations were not applied at the initial preprocessing stage because the input data already met validated operational standards.

The standardized preprocessing workflow included correction of mislabeled samples, removal of duplicates, harmonization of measurement units, interactive inspection of drilling logs and gas curves, validation of mud properties, bit records, and PVT data, operational normalization of flow out, and adjustment of key operational variables to align with international conventions. These procedures ensured consistency, comparability, and traceability of the data used for modeling^7^.

### Exploratory data analysis

Exploratory data analysis was performed to characterize variability, identify compositional trends, and define the internal structure of the data before supervised modeling. The analysis combined conventional gas ratios, AG composition vectors, operational variables, and expert interval labels. Particular attention was given to anomalous gas enrichments that could indicate drilling-induced alteration rather than native reservoir-fluid composition.

Key geochemical descriptors included C1/C2, C1/C3, C2/C3, iC4/nC4, iC5/nC5, wetness, balance, and character ratios, together with C2-, C2C-, and ETHE-derived indicators. These descriptors were used to compare intervals, screen outliers, and support the definition of affinity- and DBM-related targets. Classical mud-gas interpretation indices were calculated following established light-hydrocarbon interpretation practices^9^. This stage was designed to provide a transparent and reproducible diagnostic layer before machine-learning model training, not to replace expert interpretation.

Dimensionality-reduction and unsupervised pattern-discovery techniques were used to examine latent structure and compositional similarity within the gas datasets. Multidimensional Scaling projected the high-dimensional AG composition vectors and drilling-related parameters into a lower-dimensional space while preserving pairwise dissimilarities. K-means clustering was then applied to standardized gas-composition ratios to identify clusters representing distinct compositional regimes. These analyses supported feature selection, outlier screening, and interval-label refinement (Supplementary Fig. S1).

### Similarity analysis

To quantify how closely AG chromatographic profiles resembled laboratory-based PVT compositions, we defined a depth-dependent similarity score, S(z), from the Pearson correlation coefficient calculated over the shared light-hydrocarbon set H = {C1, C2, C3, iC4, nC4, iC5, nC5}.

For each depth z, the Pearson correlation coefficient was calculated as:

1 $$\:r\left(z\right)=\frac{{\sum\:}_{i=1}^{n}\left({G}_{AG,i}\left(z\right)-{\bar{G}}_{AG}\left(z\right)\right)\left({G}_{PVT,i}-{\bar{G}}_{PVT}\right)}{\sqrt{{\sum\:}_{i=1}^{n}{\left({G}_{AG,i}\left(z\right)-{\bar{G}}_{AG}\left(z\right)\right)}^{2}{\sum\:}_{i=1}^{n}{\left({G}_{PVT,i}-{\bar{G}}_{PVT}\right)}^{2}}}$$

Where:

* n* is the set of hydrocarbons H,

$\:{G}_{AG,i}\left(z\right)$ is the AG gas concentration at depth * z*,

$\:{G}_{PVT,i}$ is the laboratory PVT composition,

and the overbars denote means.

The resulting coefficient r(z), bounded between − 1 and + 1, was linearly transformed to S(z) = [r(z) + 1]/2, producing a normalized similarity target between 0 and 1. An uncertainty index U(z) was defined as the normalized absolute deviation between predicted similarity and reference similarity:

2 $$\:U\left(z\right)=\frac{\left|{S}_{pred}\left(z\right)-{S}_{ref}\left(z\right)\right|}{\mathrm{ma}{\mathrm{x}}_{z}\left|{S}_{pred}\left(z\right)-{S}_{ref}\left(z\right)\right|}$$

A corresponding reliability index was calculated as

3 $$\:R\left(z\right)=1-U\left(z\right)$$

clipped to the interval [0,1]. This formulation provided a consistent way to compare model outputs with reference AG-PVT similarity and to visualize lower-confidence intervals. The similarity analysis supported the construction of supervised targets for the affinity models^1,4,10^(Fig. 3).

### Machine-learning modeling

The machine-learning workflow was designed to predict two operationally relevant outputs: Reservoir Affinity Curves, which represent depth-dependent similarity between AG readings and laboratory PVT compositions, and a DBM Severity Curve, which quantifies DBM-induced alteration using ethylene behavior and drilling parameters.

The modeling pipeline included definition of target labels from AG-PVT similarity and ETHE profiles, feature selection integrating geochemical and operational signatures, KRR modeling, well-level dataset splitting to avoid data leakage, and generation of depth-dependent predictions. The models were trained using three depth-dependent target variables derived from AG-PVT similarity analysis and DBM-related ethylene behavior. These targets represented two modeling objectives: reservoir-fluid affinity and drill-bit-induced thermal alteration.

Input features were selected to represent geochemical behavior and operational conditions. They included routine and advanced gas measurements (C1-C5 and AG channels), drilling mechanics (rate of penetration, WOB, RPM, and torque), mud properties (density, salinity, and Marsh viscosity), mechanical energy indicators, and categorical descriptors such as bit model and mud type (Supplementary Table S1). KRR was selected for its ability to model nonlinear depth-dependent patterns while maintaining robustness through L2 regularization^8^.

To reduce spatial and operational leakage, data were split at the well level: 80 wells (approximately 77%) were used for training and 24 wells (approximately 23%) were reserved for independent testing. This design ensured that depth samples from the same well were not simultaneously represented in training and test subsets, reducing optimistic performance estimates caused by spatial correlation and repeated operational patterns within individual wells.

Learning curves were computed for KRR and XGBoost using well-level cross-validation. For each model, the training set was progressively subsampled, and mean training and validation scores were computed across folds. Variability envelopes (+/- 1 standard deviation) were generated to quantify model stability across training-set size (Fig. 6).

### Use of AI-assisted language editing

During manuscript revision, ChatGPT (OpenAI) was used only to assist with language editing, grammar, clarity, and formatting consistency in American English. The tool was not used to generate research data, perform statistical analyses, create figures, define the methodology, interpret results independently, or draw scientific conclusions. All AI-assisted edits were reviewed, verified, and approved by the authors, who take full responsibility for the content of the manuscript.

## Conclusion

This study presents a structured workflow that integrates real-time gas acquisition, compositional similarity analysis, and machine-learning modeling to improve fluid interpretation during hydrocarbon exploration drilling. The WebMudlogging platform supported data integrity, multi-domain harmonization, and expert validation, enabling AG measurements to be linked with laboratory PVT reference compositions. Because PVT analysis is not available in real time, the proposed approach uses laboratory data for model calibration and validation while generating depth-dependent predictive products that support interpretation during drilling.

The workflow generated three operational outputs: C2-based and C2C-based Reservoir Affinity Curves and an ETHE-based DBM Severity Curve. Together, these outputs help distinguish representative formation-fluid signatures from intervals affected by drilling-induced artifacts, especially those associated with DBM. KRR was selected as the primary deployment model because it provided stable, smooth, and interpretable predictions, whereas boosting-based models showed strong benchmark accuracy and should be evaluated further in future implementations.

By converting mud-gas signatures into quantitative affinity and DBM-severity estimates, the methodology can support fluid-sampling decisions, risk mitigation, and early reservoir characterization before laboratory results become available. Future work should validate the models prospectively in additional geologic settings, integrate predictive curves with petrophysical logs and MDT/wireline sampling data, and develop real-time dashboards with uncertainty visualization and model-monitoring capabilities. Overall, this work contributes a scalable, field-oriented framework for transforming raw mud-gas data into actionable fluid intelligence during exploration drilling.

## Supplementary Information

Below is the link to the electronic supplementary material.


Supplementary Material 1


## Data Availability

The datasets generated and analyzed during the current study are not publicly available because they contain confidential industrial information and are subject to nondisclosure restrictions. Access is limited to authorized technical staff within the company’s exploration department. Data may be made available by the corresponding author upon reasonable request and subject to institutional approval, confidentiality review, and removal of proprietary information.

## Code availability

The modeling workflow was implemented in Python using open-source libraries including scikit-learn, pandas, NumPy, Matplotlib, XGBoost, and LightGBM. Custom scripts used for data preparation and model training depend on proprietary database structures and cannot be publicly released with the manuscript. Access to non-confidential code components may be requested from the corresponding author, subject to institutional approval and removal of proprietary information.
