# Supplementary material for: Enhancing operational decision-making in hydrocarbon exploration drilling using machine learning for gas data interpretation
Source: Sci Rep. 2026 Jun 13;16:27000. doi: 10.1038/s41598-026-57038-8 (PMC13521912; doi:10.1038/s41598-026-57038-8)
Supplement: Supplementary file 1 [file 41598_2026_57038_MOESM1_ESM.pdf]

Supplementary Information

Enhancing Operational Decision-Making in Hydrocarbon Exploration Drilling Using Machine Learning for Gas Data Interpretation

Gil Marcio Avelino Silva et al.

Supplementary Figure S1

Attribute relationships visualized using Multidimensional Scaling (MDS). The plot highlights similarity relationships among gas-composition attributes, supporting dimensionality reduction and feature selection for clustering analysis.

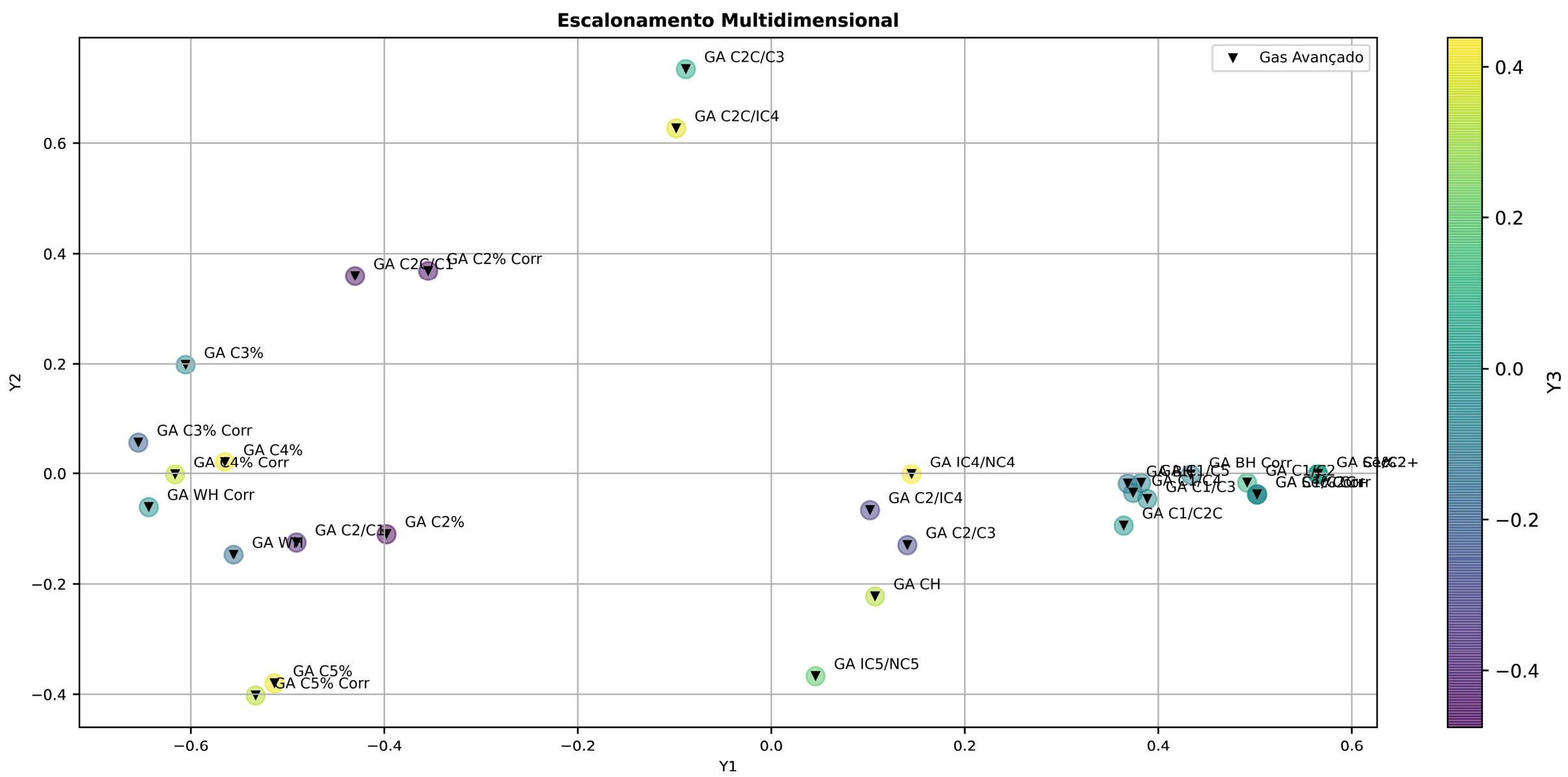

Supplementary Table S1

Input features used in training the Reservoir Affinity and DBM Severity models.

Label	Unit	Description
ROP	m/min	Rate of penetration
TORQUE	lbf-ft	Torque
RPM	rpm	Revolutions per minute
WOB	klbf	Weight on bit
FLWOUT	%	Flow out
GC-C1	ppm	Methane
GC-C2	ppm	Ethane
GC-C3	ppm	Propane
GC-IC4	ppm	Iso-butane
GC-NC4	ppm	Normal-butane
GC-IC5	ppm	Iso-pentane
GC-NC5	ppm	Normal-pentane

GC-TGAS	ppm	Total gas
GA-C1	ppm	Advanced Gas methane
GA-C2	ppm	Advanced Gas ethane plus ethylene
GA-C2C	ppm	Advanced Gas ethane
GA-C3	ppm	Advanced Gas propane
GA-IC4	ppm	Advanced Gas iso-butane
GA-NC4	ppm	Advanced Gas normal-butane
GA-IC5	ppm	Advanced Gas iso-pentane
GA-NC5	ppm	Advanced Gas normal-pentane
DIAMBROCA	inches	Bit diameter
CODMODELO_enc	categorical	Bit model
FLUIDO_enc	categorical	Mud type
DENSIDADE	g/cm3	Mud density
RAZAO_OLEO	ratio	Oil ratio
RAZAO_AGUA	ratio	Water ratio
NACL	ppm	Salinity
VISCOSIDADE_MARSH	s	Marsh funnel viscosity
MSE	lbf/in2	Mechanical specific energy
